# Tensile-Tearing Fracture Analysis of U-Notched Spruce Samples

**DOI:** 10.3390/ma15103661

**Published:** 2022-05-20

**Authors:** Ali Reza Torabi, Sobhan Mohammadi, Behnam Saboori, Majid Reza Ayatollahi, Sergio Cicero

**Affiliations:** 1 Fracture Research Laboratory, Faculty of New Sciences and Technologies, University of Tehran, Tehran 14395-1561, Iran; sobhan.mohammadi.cse@gmail.com; 2 Center of Excellence in Experimental Solid Mechanics and Dynamics, Fatigue and Fracture Research Laboratory, School of Mechanical Engineering, Iran University of Science and Technology, Tehran 16846, Iran; b_saboori@alumni.iust.ac.ir (B.S.); m.ayat@iust.ac.ir (M.R.A.); 3 LADICIM, Departamento de Ciencia e Ingeniería del Terreno y de los Materiales, Universidad de Cantabria, 39005 Santander, Spain

**Keywords:** fracture, spruce, U-notch, orthotropic materials, mixed-mode I/III loading

## Abstract

Spruce wood (*Picea Mariana*) is a highly orthotropic material whose fracture behavior in the presence of U-shaped notches and under combined tensile-tearing loading (so-called mixed-mode I/III loading) is analyzed in this work. Thus, several tests are carried out on U-notched samples with different notch tip radii (1 mm, 2 mm, and 4 mm) under various combinations of loading modes I and III (pure mode I, pure mode III, and three mixed-mode I/III loadings), from which both the experimental fracture loads and the fracture angles of the specimens are obtained. Because of the linear elastic behavior of the spruce wood, the point stress (PS) and mean stress (MS) methods, both being stress-based criteria, are used in combination with the Virtual Isotropic Material Concept (VIMC) for predicting the fracture loads and the fracture angles. By employing the VIMC, the spruce wood as an orthotropic material is modeled as a homogeneous and isotropic material with linear elastic behavior. The stress components required for calculating the experimental values of notch stress intensity factors are obtained by finite element (FE) analyses of the test configuration using commercial FE software from the fracture loads obtained experimentally. The discrepancies between the experimental and theoretical results of the critical notch stress intensity factors are obtained between −12.1% and −15% for the PS criterion and between −5.9% and −14.6% for the MS criterion, respectively. The discrepancies related to fracture initiation angle range from −1.0% to +12.1% for the PS criterion and from +1.5% to +12.2% for the MS criterion, respectively. Thus, both the PS and MS models have good accuracy when compared with the experimental data. It is also found that both failure criteria underestimate the fracture resistance of spruce wood under mixed-mode I/III loading.

## 1. Introduction

The production of components and structures made from orthotropic materials is increasing in various industries, such as the aerospace industry. On the other hand, the existence of discontinuities in real components and structures is inevitable for design purposes, such as cutouts and access doors, etc. U- and V-shaped notches are two types of prevalent notches. Notch fracture mechanics deal with discontinuities by analyzing the fracture behavior of notched components. The precise prediction of fracture conditions is a vital aspect of the corresponding design and development processes and is thus the main subject of fracture mechanics. Given the dependency of the material properties upon direction, fracture analyses in orthotropic materials are more complicated than those performed in isotropic materials.

Furthermore, the presence of notches with different geometries in structures and components is inevitable, something that can lead to severe stress concentration in the vicinity of the notch and, finally, damage and crack initiation from the notch edge. The propagation of the nucleated crack can eventually result in the catastrophic failure of the notched structure. The geometry of the notch, the material properties, and the loading conditions can affect the fracture behavior of notched components, and many researchers have investigated all these aspects from different points of view. Below, a brief literature review is performed on the fracture analysis of orthotropic materials, focusing on loading modes, analysis approaches, and the materials being investigated.

Zappalorto and Salviato [1] computed analytically the stress distribution in orthotropic plates under out-of-plane shear for linear elastic materials in four geometric configurations. In addition, Zappalorto [2] conducted an experimental study on the notch sensitivity in orthotropic materials. Torabi and Pirhadi [3] studied, by using conventional brittle fracture models, the last-ply-failure (LPF) load of composite specimens with V-notches loaded under mixed-mode I/II. Asadi et al. [4] analyzed the stress intensity factors (SIFs) for a cracked orthotropic functionally graded material (FGM) under out-of-plane shear loading. Moreover, Kumar et al. [5] investigated the crack propagation in orthotropic plates under thermal-mechanical loading using the extended finite element method (XFEM). Toktas and Dag [6] investigated oblique surface cracking in orthotropic components. Tankasala et al. [7] studied the crack propagation variations in an orthotropic solid under pure mode I loading. In addition, Chalivendra [8] calculated the stress distribution for inhomogeneous orthotropic materials for different combinations of shearing and tearing modes, employing the asymptotic analysis of Westergard’s stress functions. Phan et al. [9] studied the bending fracture of spruce wood under mixed-mode I/II loading and provided a criterion which was valid for the entire R-curve spectrum.

Mehri Khansari et al. [10] provided a micromechanical criterion for the damage zone in composite materials under mixed-mode I/II loading. Furthermore, Zare Hossein Abadi et al. [11] provided a general criterion for the fracture of orthotropic materials under mixed-mode I/II loading, including the T-stress. They showed that in an orthotropic material, the mode I critical-stress-intensity factor (K_Ic_) could not be considered as mode I resistance and that, under certain conditions, the critical-stress-intensity factor could be larger than K_Ic_. Additionally, Manafi Farid and Fakoor [12] provided a new theory for the fracture of cracked orthotropic composite components.

Fakoor and Ghoreishi [13] conducted a comprehensive survey on stress intensity factors in rotating disks having semi-elliptical cracks. Moreover, Fakoor and Khansari [14] proposed a general criterion, based on linear elastic fracture mechanics (LEFM), for a fracture analysis of orthotropic materials under mixed-mode I/II loading for the case of the misalignment of fibers with respect to the loading direction. Tschegg et al. [15] studied the mixed-mode I/II fracture of spruce wood. Gupta et al. [16] conducted a survey on the effects of T-stress on fracture, showing that this parameter has a significant influence on the crack-tip stress distribution. Fakoor and Shahsavar [17] published a review paper on the fracture of composite materials under mixed-mode I/II loading, revealing that the theories based on the linear elastic fracture mechanics (LEFM) predict fracture conditions conservatively, and do not provide accurate predictions of the fracture conditions of orthotropic materials.

Researchers have employed diverse approaches for assessing notch fractures. Mirsayar and Hartel [18] studied the fracture of notched components made of shape memory alloy components based on the strain energy density (SED). Several researchers also investigated notch fracture mechanics relying on the SED approach [19,20,21]. Some researchers studied notch fracture based on J-integral and energy methods [22,23]. SED was also employed to estimate the brittle fracture of notched components under mixed-mode loading conditions [24,25,26,27,28]. Berto [29] evaluated the effectiveness of SED in the elasto-static stress field around V-notches under mode I loading. Meneghetti et al. [30] generalized the SED criterion to mode I/III loading conditions. Other approaches such as stress-based models, finite fracture mechanics (FFM), and the Virtual Isotropic Material Concept (VIMC) have also been utilized [31,32,33,34,35,36].

Some researchers studied SIFs in an orthotropic medium using the singular integral equation and the R-curve method [37,38]. U-notches subjected to mixed-mode I/II loading were analyzed in fracture conditions in references [39,40]. In references [41,42,43] the analyses were focused on mixed-mode I/III loading cases. Berto et al. [44] assessed the pure mode III fracture of polymethyl-methacrylate (PMMA), proving the high accuracy of the SED approach. Torabi and Shahbazian [45] semi-analytically calculated the plastic region size of U-notched components under mixed-mode I/II loading. The effects of some parameters such as plasticity phenomena, inertia, and T-stress on fracture were studied in references [46,47]. Mixed-modes I/III and I/II fractures of epoxy adhesive, Al 2034, and wood were evaluated in references [48,49,50,51]. Razavi and Berto [52] proposed a new fixture for fracture testing under mixed-mode I/II/III loading. Chuchala et al. [53] determined the critical-stress-intensity factor and shear yield stress for pine and beech woods based on the cutting machine’s power signal. In addition, they investigated four different drying methods and showed that the drying method has a meaningful effect on the critical-stress-intensity factor and shear yield stress. Wyeth et al. [54] conducted a series of tests on Douglas fir wood on two low- and high-speed rigs with the aim of understanding the relation between chip formation, critical-stress-intensity factor, and resistance forces, and the work required for cutting. They argued that a distinction has to be made regarding the way the chip is formed by shear or by bending. Sinn et al. [55] studied the effect of the machining process on the surface characteristics of wood, such as roughness, color, wettability, and surface free energy. The mixed-mode I/II fracture of wood was explored in references [56,57,58]. Additionally, in references [59,60], the fracture of orthotropic specimens was analyzed using the peridynamics theory. Moreover, references [61,62,63] utilized the boundary element method (BEM) and XFEM for fracture parameter extraction. Meanwhile, references [64,65,66] investigated the fracture behavior of spruce wood under combined loading conditions.

With all this, the aim of the present study is the assessment of U-notched orthotropic components under mixed-mode I/III loading in the linear elastic regime. U-notched spruce wood specimens that have three different values of notch tip radii are tested under five different mixed-mode loading cases. Experimentally acquired fracture loads are applied to the finite element (FE) models of the test configuration to compute the critical-notch-stress-intensity factor values calculated based on the related stress components. Then, both the points stress (PS) and the mean stress (MS) criteria are used to predict fracture conditions theoretically. Both criteria are the stress-based fracture models developed based on LEFM and they have already been utilized in diverse studies investigating the mixed-mode I/II brittle fracture of notched components successfully (see, for instance, reference [67]). Although both criteria have been developed based on isotropic elasticity assumptions, their combination with VIMC is used in this paper to study the fracture behavior of U-notched specimens made of spruce wood, which is an orthotropic material. The satisfactory agreement between the theoretical results and the experimental data will be shown below.

## 2. Materials and Methods

### 2.1. Experimental Programme

**Figure 1:**
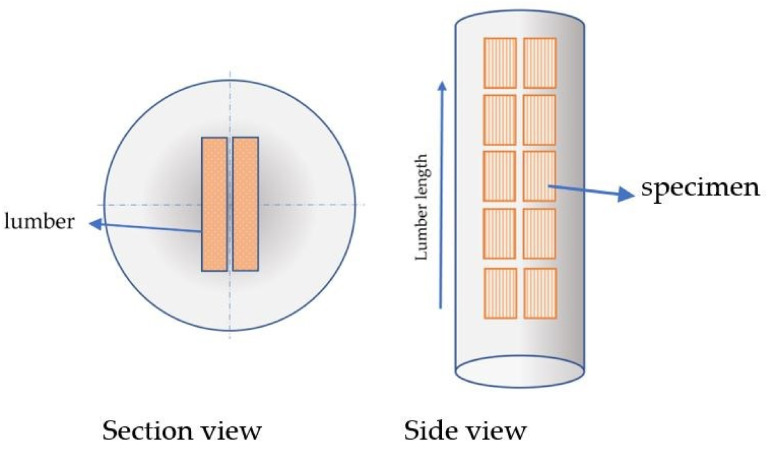
Specimen production scheme.

All test specimens were made of spruce wood (*Picea Mariana*), which has basically an orthotropic structure. The specimens were fabricated with a high-precision water-jet machine from lumbers produced from the core segment of a tree. All the specimens were cut in such a way the wood fibers are aligned with longitudinal direction of test specimens, as shown in Figure 1. Care was taken that the specimens had no natural defects of wood in them, particularly in the vicinity of the pre-existing cracks and notches.

**Figure 2:**
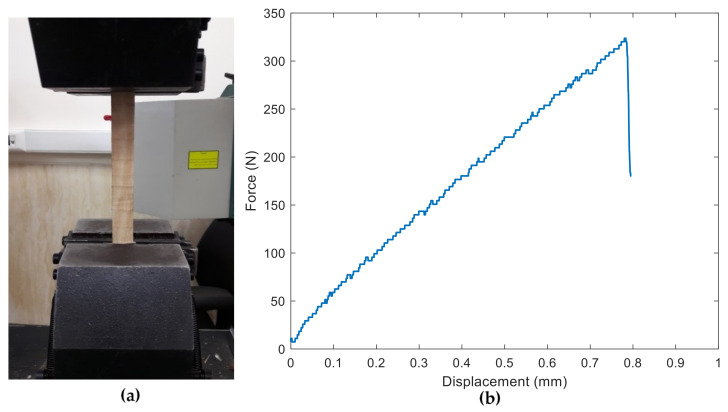
(**a**) example of tensile test configuration; (**b**) example of force-displacement curve obtained in a tensile test of spruce wood.

The lumbers were conditioned at 20 °C and 65% relative humidity (RH) until equilibrium, which implies 14% moisture content of the specimens based on weight measurement before and after dried state of wood. First, the tensile properties were obtained by loading the wood along the direction of the fibers (i.e., longitudinally). Three tensile tests were performed on rectangular specimens (see Figure 2a). Figure 2b illustrates the force-displacement behavior of spruce wood obtained from a tensile test. As the figure shows, the wood being analyzed clearly exhibits a linear behavior in the direction parallel to the fiber, which is one of the main assumptions of the present research. The main resulting tensile properties are listed in Table 1.

**Figure 3:**
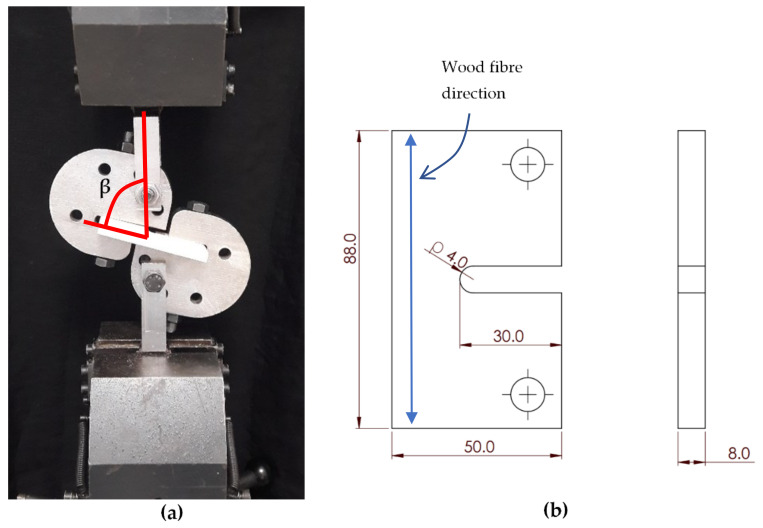
(**a**) Fracture test configuration and *β* angle definition; (**b**) U-notched test specimen (dimensions in mm), with *ρ* = 4.0 mm.

**Figure 4:**
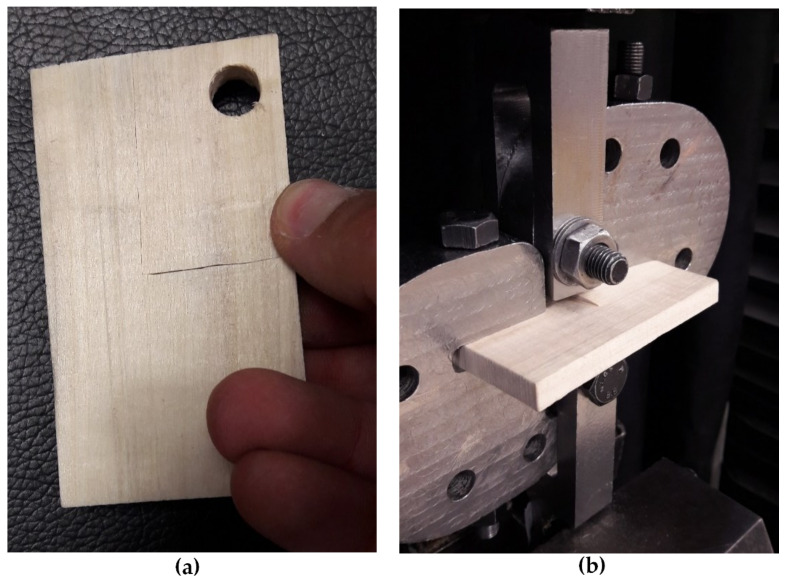
(**a**) *K_Ic_* test specimen after fracture; (**b**) K_IIIc_ test specimen during loading.

A series of fracture experiments were conducted on U-notched specimens made of spruce wood by using the test configuration shown in Figure 3a in order to obtain the notch resistance of the spruce under mixed-mode I/III loading conditions. This test setup had been previously employed successfully for the evaluation of fracture processes in both U-notched components [68,69] and cracked components [70,71] made of isotropic materials. The notch radii (*ρ*) took values of 1 mm, 2 mm, and 4 mm. The geometric details of a U-notched specimen with a notch tip radius of 4 mm are seen in Figure 3b. As observed in Figure 3a, the test specimen is fastened to each half of the loading fixture by means of a bolt and nut. The fixture creates mixed-mode I/III loading conditions by changing the specimen orientation with respect to the loading direction. Therefore, as depicted in Figure 3a, *β* angle is defined as the angle between the loading direction and longitudinal direction of the specimen. By increasing *β* from zero to 90°, the portion of mode I loading decreases and that of mode III increases. In particular, *β* = 0° and *β* = 90° correspond, respectively, to pure mode I and pure mode III loading cases. Additionally, fracture tests were performed on cracked specimens (for consistency, same geometry as that shown in Figure 3b, with *ρ* ≈ 0) in order to determine the material critical-stress-intensity factor in mode I (*β* = 0°) and mode III (*β* = 90°) loading conditions. Three tests were performed per loading condition, with Figure 4 showing two examples. The cracks were generated by sawing using a razor blade. The results are gathered in Table 1.

In order to evaluate the effects of the notch geometry on the fracture characteristics, the U-notched wood specimens were made with the three different notch tip radii mentioned above (1 mm, 2 mm, and 4 mm). For each series of the specimens with identical notch tip radii, fracture tests were performed under pure mode I (*β* = 0°), pure mode III (*β* = 90°), and three mixed-mode I/III loadings (*β* = 40°, 65°, and 72°). In addition, each specific test case was repeated three times. After installing the specimens inside the fixture, a tensile force with a constant rate of 1 mm/min was applied to the fixture by means of a tensile loading machine. The load and displacement data were recorded during the tests. The tests were continued up to the rupture of the test samples, which corresponds to the onset of a sudden drop of the force in the force-displacement diagram.

**Figure 5:**
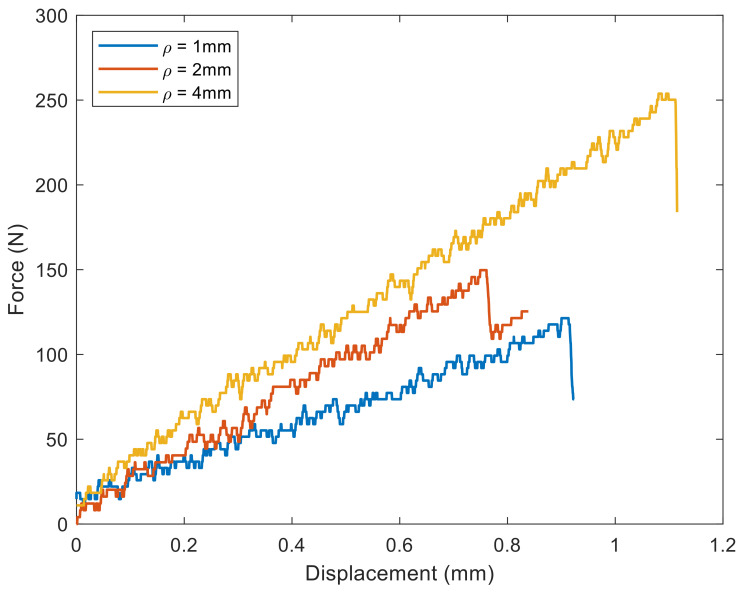
Example of force-displacement curves obtained in fracture tests of spruce wood (*β* = 40°).

The fracture loads of all U-notched specimens tested are tabulated in Table 2. Additionally, as an example, the force-displacement curves of U-notched specimens with three different tip radii fractured under *β* = 40° mixed-mode loading are plotted in Figure 5. The notch effect is evident, with an increase in the fracture load when the notch radius increases.

### 2.2. Finite Element Modeling

In order to compare the results of the fracture experiments with the predictions of the two theoretical criteria (PS and MS), which are explained in the next section, it is necessary to compute the notch-stress-intensity factors (NSIFs) of the corresponding wood samples. Thus, finite element (FE) models of the test assembly consisting of the loading fixture and the specimen were created to obtain the values of the normal and out-of-plane (anti-plane) shear stresses at the notch tip. This section describes the FE modeling of the test assembly.

The FE modeling and the subsequent analysis were carried out using the ABAQUS V6.14 commercial software package. The FE model of the test assembly consists of two halves of the loading fixture, connecting bolts, and test specimen. The contacts between the mentioned parts were defined in the modeling because of their importance in the stress calculation. More precisely, the contact between the connecting bolts and the specimen was modeled by means of the surface-to-surface contact model of the ABAQUS FE software. The same contact type was considered for the contact between the loading fixture and the connecting bolts. In all of the contacts modeled, the effect of friction was neglected because of the relatively small looseness of the specimens when they are fitted in the fixture. Furthermore, the deformation of the bolts was disregarded and they were modeled as rigid bodies since they were made from high-strength steel and, accordingly, their stiffness was one order of magnitude higher than that of the spruce wood. Finally, although the spruce wood is orthotropic in nature, it was modeled as a homogeneous and isotropic material with linear elastic behavior, thanks to the application of the Virtual Isotropic Material Concept (VIMC) [30,31,32,33,34,35].

**Figure 6:**
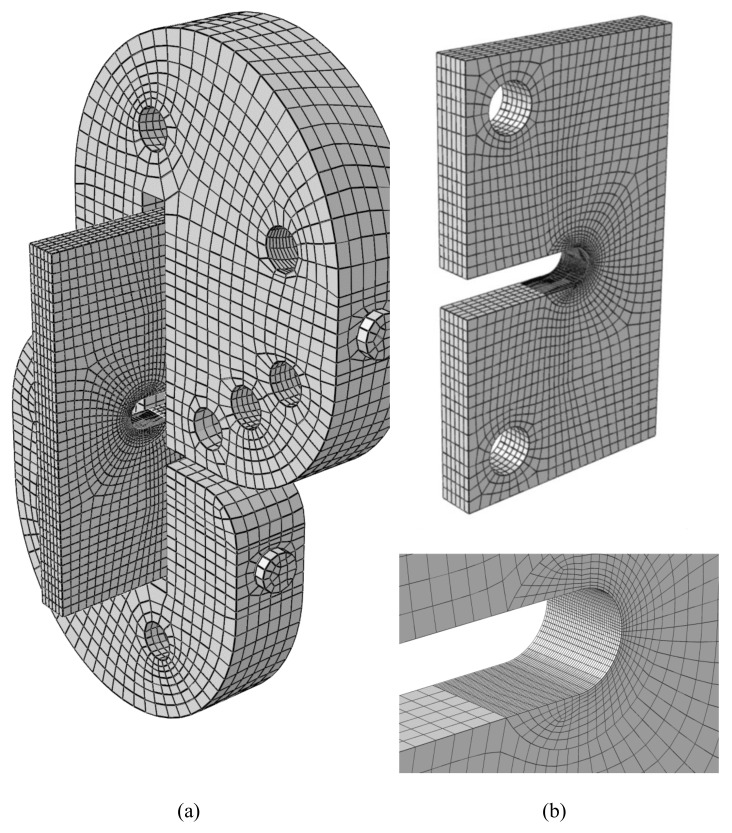
Finite element model. (**a**) Fixture and specimen configuration; (**b**) meshed specimen.

Appropriate meshing is of significant importance in all FE analyses, particularly in fracture analyses. One of the FE models is shown in Figure 6. The specimen is meshed by using brick elements with 20 nodes, all of them having three translational degrees of freedom (DOF). The high-stress gradient existing at the notch tip and its neighboring regions makes the refining of the elements necessary. Figure 6b depicts the structure and the size of the mesh used for the specimen. The minimum element size considered in the notch vicinity is 0.2 mm. Here, it is important to note that the element size of the specimen FE model was determined based on a mesh convergence analysis.

### 2.3. Fracture Criteria

During the last few decades, different approaches have been developed for the fracture analysis of isotropic engineering materials weakened by notches, such as stress/strain-based models, the cohesive zone model (CZM), finite fracture mechanics (FFM), and some others. Recently, some of these approaches have also been utilized for the last-ply-failure prediction of polymer matrix composites (PMCs), which are actually categorized as orthotropic materials [30,31,32,33,34,35]. The use of failure criteria of isotropic materials for the critical load prediction of orthotropic materials has been applied by equating the real orthotropic material with a virtual isotropic material using the Virtual Isotropic Material Concept (VIMC) [30,31,32,33,34,35]. For the sake of brevity, VIMC is not explained herein and the readers of this manuscript are referred to references [30,31,32,33,34,35]. Here, suffice it to say that the main assumption of the VIMC is that it equates a real material with orthotropic behavior to a virtual brittle material of the same geometry with isotropic behavior. Once this is done, if the VIMC is correct, well-known fracture methodologies (e.g., PS, MS) can be directly applied. Additionally, the VIMC requires (exclusively) two important properties of the orthotropic material to be defined: the ultimate tensile strength (*σ_u_*) and the critical-stress-intensity factor (*K_c_*). The accurate definition of these two parameters is the key concern of the application of the VIMC. Once they are known, they are considered as the toughness and the strength of the virtual isotropic material, and the fracture assessment of the orthotropic material being analyzed follows the same methodologies as those used for isotropic materials. In this case, the tests described above (Section 2.1) provide the mechanical properties (see Table 1) of the virtual isotropic material [30,31,32,33,34,35].

Two stress-based brittle fracture criteria, the point stress (PS) method and the mean stress (MS) method, are used in the present research. The two aforementioned criteria have already been utilized in diverse studies investigating the mixed-mode I/II brittle fracture of components containing round-tip V-notches (e.g., reference [67]). As described below, both the PS and the MS models require knowing the ultimate tensile strength and the critical-stress-intensity factor of the (linear elastic) material being analyzed, in order to derive the corresponding estimation of fracture (critical) loads. These values, for the spruce wood analyzed in this work, are gathered in Table 1.

**Figure 7:**
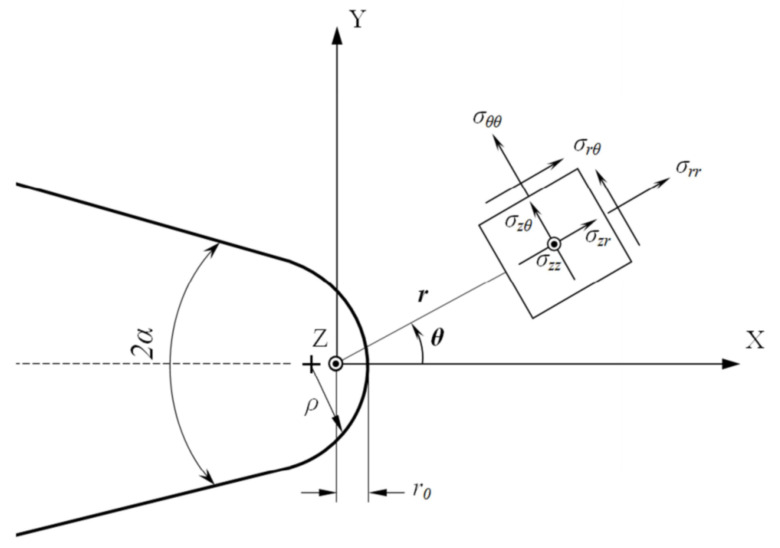
Typical blunt V-notch with its coordinate system and the resulting geometrical parameters.

The coordinate system used for the formulation of the two criteria is presented in Figure 7, which also shows a typical blunt V-shaped notch. The PS criterion assumes that fracture initiates from a point on the notch round edge where the tangential stress is maximum. This criterion also supposes that at fracture onset, the tangential stress at a particular critical distance *r_c,U_* from the origin of the coordinate system reaches the critical stress of the material (*σ_c_*), which is assumed to be equal to the material ultimate tensile strength (*σ_u_*) in brittle and quasi-brittle materials. The MS criterion, however, assumes that fracture takes place when the mean tangential stress over a particular critical distance *d_c,U_* reaches *σ_c_*. Details of the MS and PS criteria can be found, for example, in a previously published paper [72].

Using the PS and MS criteria, it is possible to generate a curve known as the fracture limit curve, which depends on the notch geometric parameters, such as the notch tip radius and opening angle. The fracture limit curve represents the fracture resistance of notched specimens with a specific geometry under different combinations of tension and out-of-plane shear loading. The fracture limit curves obtained from either the PS or MS criterion are similar and their difference is usually not significant. Both criteria require the mode I critical-notch-stress-intensity factors, *K_Ic_^U,ρ^*, for generating the fracture limit curve. *K_Ic_^U,ρ^* is obtained experimentally and it is not a material constant, since it does not only depend on the material, but also on the geometry (e.g., notch tip radius).

Given that, from a geometrical point of view, a U-notch is a blunt V-shaped notch with the opening angle of *2α* = 0°, the PS criterion determines the normalized NSIFs, *(K_III_^U,ρ^)*⁄*(K_Ic_^U,ρ^)* and *(K_I_^U,ρ^)*⁄*(K_Ic_^U,ρ^)*, per each mode mixity ratio by solving the following equations simultaneously [73]:

(1) $$\frac{K_{I}^{U,\rho }}{r_{c,U}{}^{S}}\left[\nu \left(L\left(A+R\right)+M{\left(\frac{r_{c,U}}{r_{0}}\right)}^{P}\left(B+V\right)\chi_{d1}\right)-L\left(R+S\chi_{b1}\right)-M{\left(\frac{r_{c,U}}{r_{0}}\right)}^{P}\left(\chi_{c1}+V\chi_{d1}\right)\right]\sin2\phi_{f}\;-\frac{2K_{III}^{U,\rho }}{r_{c,V}{}^{K}}\left[1+{\left(\frac{r_{c,U}}{r_{3}}\right)}^{Z}\right]\cos2\phi_{f}=0$$

(2) $$\frac{K_{I}^{U,\rho }}{K_{Ic}^{U,\rho }}\left[L\left(R+S\chi_{b1}\right)+M{\left(\frac{r_{c,U}}{r_{0}}\right)}^{P}\left(\chi_{c1}+V\chi_{d1}\right)\right]\cos^{2}\phi_{f}-\frac{K_{III}^{U,\rho }}{K_{Ic}^{U,\rho }}r_{c,U}{}^{\lambda_{3}-\lambda_{1}}\left[1+{\left(\frac{r_{c,U}}{r_{3}}\right)}^{Z}\right]\sin2\phi_{f}+\nu \frac{K_{I}^{U,\rho }}{K_{Ic}^{U,\rho }}\left[L\left(A+R\right)\;+M{\left(\frac{r_{c,U}}{r_{0}}\right)}^{P}\left(B+V\right)\chi_{d1}\right]\sin^{2}\phi_{f}=L\left(R+S\chi_{b1}\right)+M{\left(\frac{r_{c,U}}{r_{0}}\right)}^{P}\left(\chi_{c1}+V\chi_{d1}\right)$$

All the parameters in Equations (1) and (2), except for *K_I_^U,ρ^*, *K_III_^U,ρ^*, *r*_0_, *r_c,U_*, *υ*, and *ϕ_f_*, are constant coefficients which depend on the notch opening angle, and have been introduced in reference [73]. The out-of-plane fracture angle *ϕ_f_* varies from 0 in mode I conditions up to π/4 in mode III loading conditions [73]. *R_c,U_,* as the critical radial distance from the origin of the notch polar coordinate system, is derived from Equation (3), in which *r_c_* is the critical distance measured from the notch tip and *r*_0_ is the distance between the notch tip and the origin of the coordinate systems. Note that the expression of *r*_0_ for U-notches is simply *r*_0_ = *ρ*/*2*, where *ρ* denotes the notch tip radius.

(3) $$r_{c,U}=r_{0}+r_{c}\;$$

*r_c_* can be calculated from Equation (4), which has previously been derived for brittle and quasi-brittle materials subjected to mode III loading conditions, in which *K_IIIc_* is the mode III critical-stress-intensity factor of the material [73]:

(4) $$r_{c}=\frac{1}{2\pi }{\left(\frac{K_{IIIc}}{\sigma_{u}}\right)}^{2}$$

Likewise, the values of *(K_III_^U,ρ^)*⁄*(K_Ic_^U,ρ^)* and *(K_I_^U,ρ^)*⁄*(K_Ic_^U,ρ^)* predicted when using the MS criterion can be obtained by solving the following equations [73]:

(5) $$K_{I}^{U,\rho }\left(\nu C^{*}-A^{*}\right)\sin2\phi_{f}+2K_{III}^{U,\rho }B^{*}\cos2\phi_{f}=0$$

(6) $$\frac{K_{I}^{U,\rho }}{K_{Ic}^{U,\rho }}A^{*}\cos^{2}\phi_{f}+d_{c,V}{}^{\lambda_{3}-\lambda_{1}}\frac{K_{III}^{U,\rho }}{K_{Ic}^{U,\rho }}B^{*}\sin2\phi_{f}+\nu \frac{K_{I}^{U,\rho }}{K_{Ic}^{U,\rho }}C^{*}\sin^{2}\phi_{f}=A^{*}$$

The relations calculating the constant parameters A*, B*, and C* are provided in reference [73]. In addition, the critical distance *d_c,U_* can be calculated as follows:

(7) $$d_{c,U}=r_{0}+d_{c}$$

where *d_c_* is the critical distance measured from the notch tip. To determine *d_c_* for brittle and quasi-brittle materials under the loading conditions involving mode III, Equation (8) can be applied [73].

(8) $$d_{c}=\frac{2}{\pi }{\left(\frac{K_{IIIc}}{\sigma_{u}}\right)}^{2}$$

In addition to *(K_III_^U,ρ^)*⁄*(K_Ic_^U,ρ^)* vs. *(K_I_^U,ρ^)*⁄*(K_Ic_^U,ρ^)* curves, plotting the fracture limit curves (i.e., effective NSIF, *K_eff_^U,ρ^*, vs. the mode mixity ratio *M_U_^e^*) is a useful assessment tool, as it provides an opportunity to compare the mixed-mode fracture results directly by using a unique parameter. The mode mixity ratio *M_U_^e^* and *K_eff_^U,ρ^* can be computed according to the following equations:

(9) $$M_{U}^{e}=\frac{2}{\pi }\tan^{-1}\left(\frac{K_{I}^{U,\rho }}{K_{III}^{U,\rho }}\right)$$

(10) $$K_{eff}^{U,\rho }=\sqrt{{\left(\frac{K_{I}^{U,\rho }}{K_{Ic}^{U,\rho }}\right)}^{2}+{\left(\frac{K_{III}^{U,\rho }}{K_{Ic}^{U,\rho }}\right)}^{2}}$$

## 3. Results and Discussion

In this section, the fracture resistance and the out-of-plane fracture angle predictions provided by both the PS and the MS criteria are compared with the experimental data obtained from the fracture tests of the U-notched spruce wood specimens. With this aim, the theoretical and the experimental values of the mode I and mode III NSIFs must be calculated. By applying the experimental fracture loads to the FE models of the test specimens, normal and out-of-plane shear stresses are calculated. Then, the experimental values of *K_I_^U,ρ^* and *K_III_^U,ρ^* can be determined for each case by using the following equations [73]:

(11) $$K_{I}^{U,\rho }=\sqrt{2\pi }\frac{\sigma_{\theta \theta }\left(r_{0},0\right)r_{0}{}^{1-\lambda_{1}}}{1+\omega_{1}}$$

(12) $$K_{III}^{U,\rho }=\sqrt{2\pi }\frac{\tau_{max}r_{0}{}^{1-\lambda_{3}}}{\omega_{3}}$$

where *σ_θθ_*(*r*_0_,0) is the tangential stress at the U-notch tip and *τ_max_* is the maximum elastic shear stress at the notch tip, which is determined by the following Equations (13) and (14) [34]:

(13) $$\tau_{max}=\frac{\omega_{3}\sigma_{z\theta }\left(r_{0},0\right)}{1+{\left(\frac{r_{0}}{r_{3}}\right)}^{\mu_{3}-\lambda_{3}}}$$

(14) $$r_{3}≅\left(1-\mu_{3}\right)\times \rho$$

where the value of the parameters *λ*_1_, *λ*_3_, *ω*_1_, *ω*_3_, and *μ*_3_ for U-notches are 0.40978, 0.5, 0.5, 0.34, and 2.0155, respectively [72,73,74].

**Figure 8:**
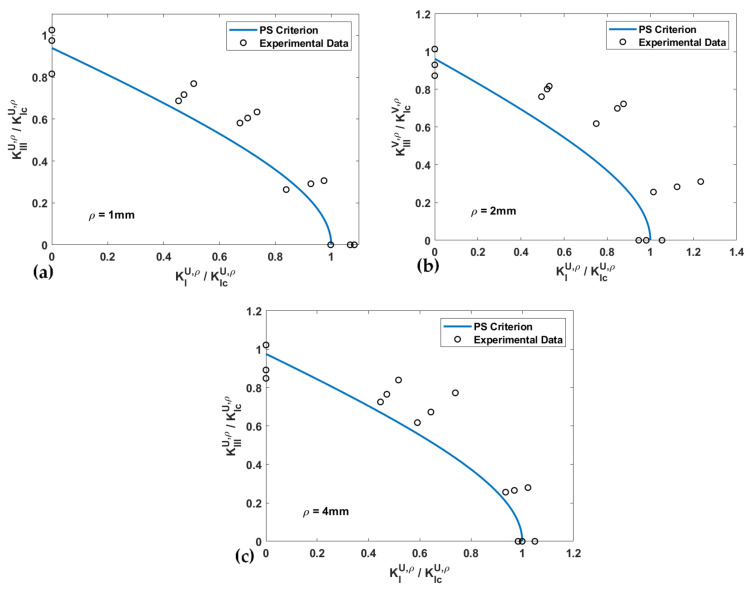
Fracture limit curves of the PS criterion and the experimental data of U-notched spruce wood specimens. (**a**) *ρ* = 1 mm; (**b**) *ρ* = 2 mm; (**c**) *ρ* = 4 mm.

**Figure 9:**
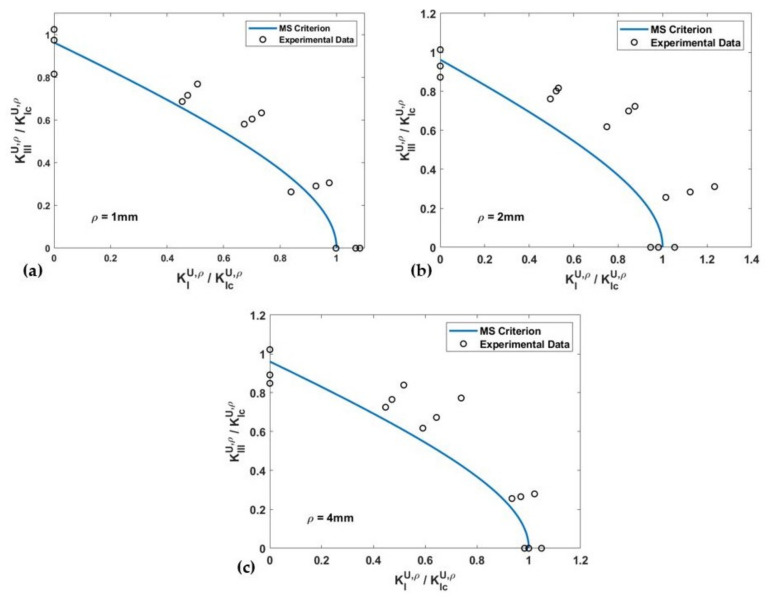
Fracture limit curves of the MS criterion and the experimental data of U-notched spruce wood specimens. (**a**) *ρ* = 1 mm; (**b**) *ρ* = 2 mm; (**c**) *ρ* = 4 mm.

The fracture limit curves, in terms of the normalized NSIFs for PS and MS criteria, and the experimental data of the U-notched spruce wood specimens for all notch tip radii, are shown and compared in Figure 8 and Figure 9, respectively. The estimated values for the effective normalized NSIF (*K_eff_^U,ρ^*) obtained when using the PS and the MS criteria, and the mean values of the experimental *K_eff_^U,ρ^*, are listed in Table 3, which also gathers the discrepancies between the experimental and the theoretical results (*∆*) for notch the radii of 1 mm, 2 mm, and 4 mm. The average discrepancies are between −12.1% and −15% for the PS criterion and between −5.9% and −14.6% for the MS criterion. One can compare the mentioned discrepancies with those reported in reference [43], which fall into an interval of ±20%.

The discrepancies show that although the PS and MS criteria are fracture models based on isotropic elasticity assumptions, they are able to predict the fracture of highly orthotropic components weakened by U-notches under mixed-mode I/III loading conditions when combined with the VIMC. On the basis of the discrepancies obtained when using the two criteria, it can be stated that neither of them is preferable to the other from the point of view of precision, although in this case, the MS criterion provides a slightly better accuracy; however, the PS criterion has less complexity and is more straightforward. Additionally, it is important to note that both the PS and the MS criteria tend to provide underestimations of the effective NSIFs.

One of the sources of the discrepancy between the two theoretical approaches and the experimental results is that both PS and MS criteria rely on an approximate stress distribution based on the isotropic theory of elasticity, which is not completely consistent with the orthotropic stress distribution. Therefore, this leads to some deviation in the predicted stress values of the specimens made of wood, which is a natural composite material with a non-isotropic structure. A second cause of the discrepancy may be that the present research relies on linear elastic assumptions, neglecting any plastic zone at the notch-edge neighborhood, which could definitely postpone fracture by absorbing some amount of energy. A third factor contributing to the discrepancies is the material utilized in the tests. Clearly, the mechanical properties of wood have a dependency on several factors, such as humidity, the natural orientation of fibers, and the existence of pre-existing defects that woods have intrinsically. Some of these factors are hardly controllable. Finally, keeping dimensional stability in a large number of test specimens is another item affecting the discrepancy between the theoretical and experimental results.

Figure 8 and Figure 9 show that except for the pure mode III loading case, both the PS and the MS criteria provide conservative estimations of the notch fracture resistance of spruce wood. While the deviation in the predictions does not demonstrate a clear relation with the notch tip radius, it can be stated that deviations are larger for the loading modes corresponding to *β* = 40° and 65°. In addition, the total average of the discrepancies reported in Table 3 suggests that the predictions of the PS criterion are slightly more conservative than those of the MS criterion.

**Figure 10:**
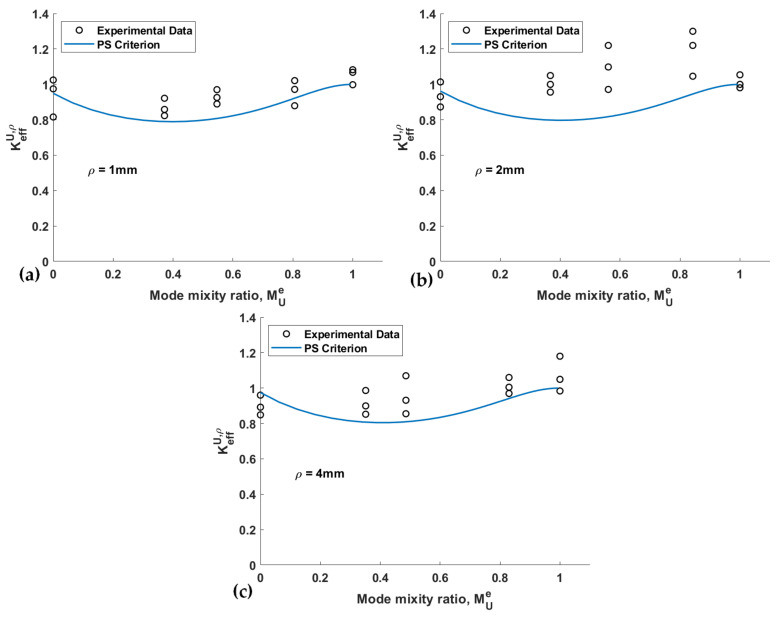
Effective normalized fracture resistance predictions vs. mode mixity ratio when using the PS criterion and corresponding experimental data. (**a**) *ρ* = 1 mm; (**b**) *ρ* = 2 mm; (**c**) *ρ* = 4 mm.

**Figure 11:**
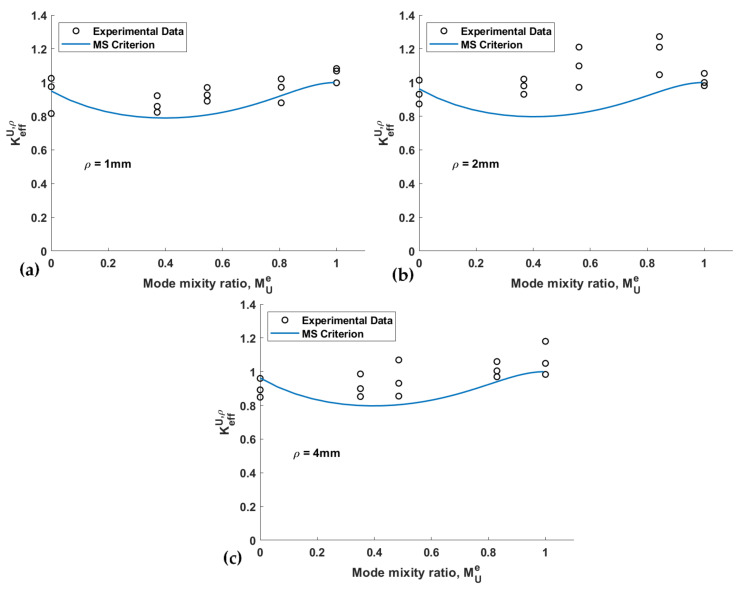
Effective normalized fracture resistance predictions vs. mode mixity ratio when using the MS criterion and corresponding experimental data. (**a**) *ρ* = 1 mm; (**b**) *ρ* = 2 mm; (**c**) *ρ* = 4 mm.

The variation of the effective normalized NSIF versus the mode mixity ratio for both the PS and the MS criteria, as well as the experimental data of the spruce wood samples, are plotted in Figure 10 and Figure 11, respectively. These figures reveal that both criteria predict an initial decrease in *K_eff_^U,ρ^* when changing the loading mode from pure mode I to mixed-mode I/III loading, with minimum values at mixity values around 0.4. After this value, there is an increase in *K_eff_^U,ρ^* until pure mode III loading conditions, where *K_eff_^U,ρ^* values are similar to those predicted for pure mode I conditions. Similar behavior for *K_eff_^U,ρ^* and mixity values has been reported by Aliha et al. [41]. The experimental data follow the same trends on certain occasions. However, in the case of the loading modes corresponding to *β* = 40° and 65°, the experimental data have larger deviations from the predictions. In other words, both fracture criteria provide more conservative predictions under mixed-mode I/III loading conditions than under pure modes I and III.

**Figure 12:**
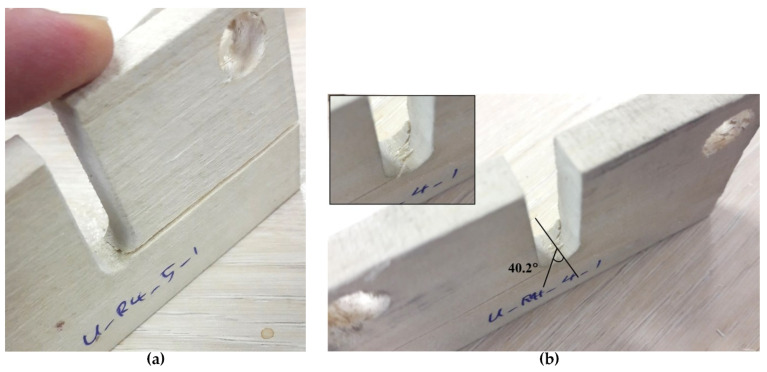
U-notched spruce wood specimens with 4 mm tip radius after failure under the loading angle of: (**a**) *β* = 0° (pure mode I); (**b**) *β* = 72°.

**Figure 13:**
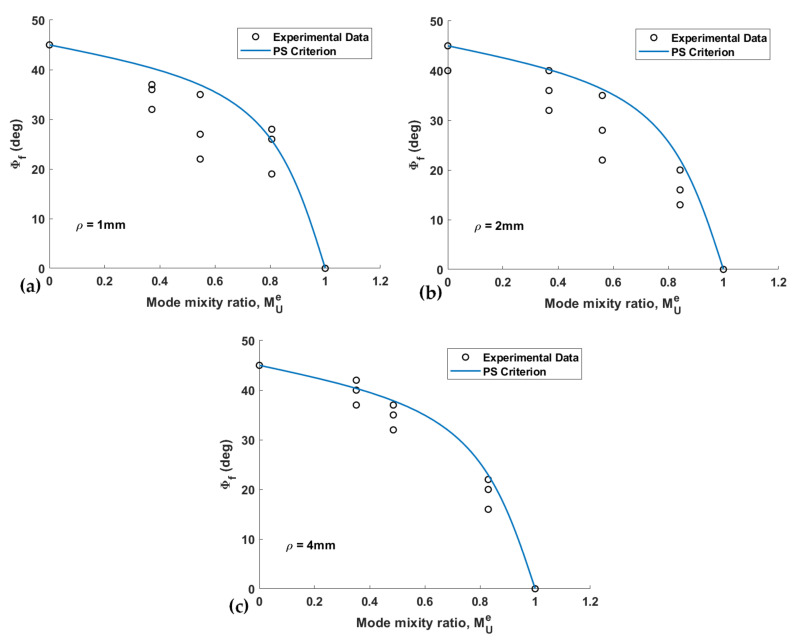
Out-of-plane fracture angle predicted by the PS criterion and corresponding experimental data. (**a**) *ρ* = 1 mm; (**b**) *ρ* = 2 mm; (**c**) *ρ* = 4 mm.

**Figure 14:**
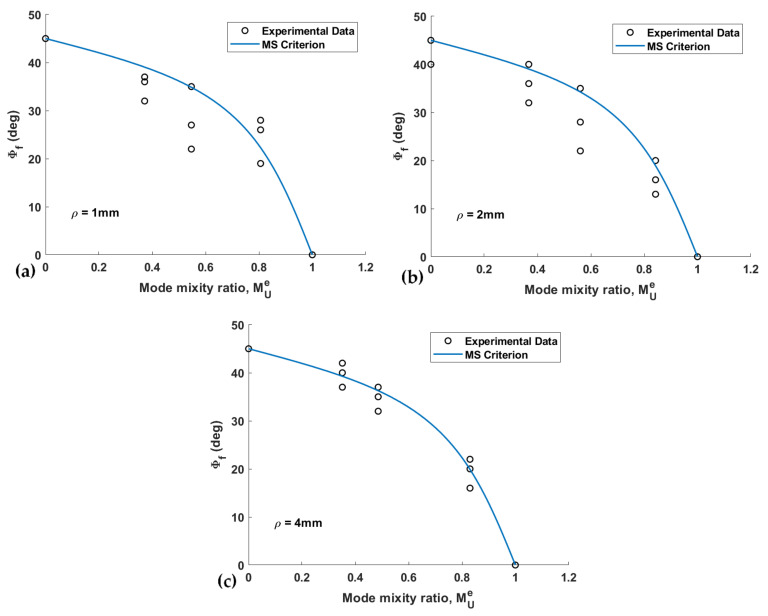
Out-of-plane fracture angle predicted by the MS criterion and corresponding experimental data. (**a**) *ρ* = 1 mm; (**b**) *ρ* = 2 mm; (**c**) *ρ* = 4 mm.

Figure 12 shows two of the tested U-notched wood specimens with *ρ* = 4 mm, which were subjected to different loading conditions. Figure 12b shows that the out-of-plane fracture angles of the specimens tested are measured from the images of the specimens after failure by means of a traditional software. Figure 13 and Figure 14 gather the out-of-plane fracture angle *ϕ_f_* predicted by the PS and MS criteria, respectively. These figures also contain the corresponding measured experimental data. The fracture angles estimated by the PS and the MS criteria are also compared with the experimental data in Table 4, providing the discrepancies (*∆*, %).

The discrepancy, with reference to the experimental results, ranges from −1.0% to +12.1% for the PS criterion and from +1.5% to +12.2% for the MS criterion, implying the satisfactory capability of both approaches for the prediction of the fracture angle. Both approaches tend to overestimate the out-of-plane fracture angle. Similar to fracture resistance, the fracture angle predictions of the two criteria are close to each other. Moreover, the minimum discrepancy for both criteria corresponds to the specimen with *ρ* = 4 mm. The main reason could be the smoothness of the stress distribution around the notch that affects the crack path to a lesser extent. It is noteworthy that the plots do not show a special relationship between the mode mixity ratio and the obtained predictions.

## 4. Conclusions

For the first time, this work analyzed the fracture of U-notched specimens made of spruce wood, which is a highly orthotropic material, subjected to mixed-mode I/III loading conditions. A series of mixed-mode I/III fracture tests (including pure mode I, pure mode III, and three mixed-mode I/III loadings) were conducted on U-notched spruce wood specimens fabricated with three different notch tip radii (1 mm, 2 mm, and 4 mm). Two stress-based theoretical fracture criteria, namely the point stress (PS) and the mean stress (MS) approaches, were employed in conjunction with the Virtual Isotropic Material Concept (VIMC) for predicting the corresponding notch fracture resistance and the out-of-plane fracture angle. The calculation of the notch-stress-intensity factors (NSIFs) of the notched specimens tested was accomplished by 3D finite element analysis. It was found that both the PS and the MS have satisfactory agreement with the experimental data in all the loading cases tested. Discrepancies between the experimental and theoretical results of the critical notch stress intensity factors were obtained between −12.1% and −15% for PS criterion and between −5.9% and −14.6% for MS criterion, respectively. The discrepancies related to fracture angle were between −1.0% and +12.1% for PS criterion and between +1.5% and +12.2% for MS criterion, respectively. No meaningful preference between the criteria was detected from the point of view of their accuracy, with the two of them providing estimations of the critical notch stress intensity factors that are generally lower than the experimental values, and out-of-plane fracture angle predictions that are mostly higher than the experimental values. This investigation clearly demonstrated that PS and MS approaches are not only appropriate for mixed-mode I/III fracture predictions of U-notched samples made of isotropic materials, but also for those made of orthotropic materials.

## Figures and Tables

**Table 1:** Mechanical properties of the spruce wood tested.

Material Properties	Mean Value	Standard Deviation
Elastic modulus, *E* [GPa]	8.35	0.75
Ultimate tensile strength (axial), *σ_u_* [MPa]	58.80	5.46
Poisson’s ratio, *ν*	0.32	0.04
Mode I critical-stress-intensity factor, *K_Ic_* [MPa√m]	0.75	0.08
Mode III critical-stress-intensity factor, *K_IIIc_* [MPa√m]	0.25	0.03

**Table 2:** Fracture loads of the U-notched spruce wood fracture specimens.

Notch Radius *ρ* (mm)	Loading Angle *β* (°)	Fracture Load (N)	Average Fracture Load (N)
1	0 (mode I)	121.2	157.3
		181.2	
		169.6	
	40	147.2	147.2
		121.4	
		172.9	
	65	172.9	176.5
		165.2	
		191.3	
	72	187.6	186.4
		176.6	
		195.0	
	90 (mode III)	140.8	169.6
		172.9	
		195.0	
2	0	257.5	272.6
		286.9	
		273.4	
	40	165.5	153.3
		136.1	
		158.4	
	65	191.3	201.1
		232.8	
		179.2	
	72	195.0	185.2
		161.9	
		198.7	
	90	143.5	167.9
		191.2	
		168.9	
4	0	323.7	308.0
		301.5	
		298.7	
	40	253.8	255.0
		242.8	
		268.5	
	65	147.2	175.8
		185.6	
		194.5	
	72	173.0	183.8
		169.2	
		209.3	
	90	228.1	212.6
		217.3	
		192.4	

**Table 3:** Theoretical and experimental values of the effective normalized NSIF for the U-notched spruce wood samples, together with the percent discrepancies.

Notch Radius *ρ* (mm)	Loading Angle *β* (°)	Mean Experimental Effective Normalized NSIF	PS Criterion		MS Criterion	
			Effective Normalized NSIF	Δ(%)	Effective Normalized NSIF	Δ(%)
1	40	1.03	0.98	−5.6	0.95	−7.8
	65	1.00	0.83	−17.1	0.87	−13.5
	72	0.94	0.75	−20.4	0.80	−15.0
	90	1.01	0.86	−14.8	1.14	+12.5
			**Avg.**	−14.5	**Avg.**	−5.9
2	40	1.07	0.95	−12.1	0.93	−13.3
	65	0.98	0.82	−16.8	0.80	−18.4
	72	0.92	0.80	−12.1	0.77	−16.1
	90	1.03	0.96	−7.3	0.92	−10.8
			**Avg.**	−12.1	**Avg.**	−14.6
4	40	1.06	0.98	−8.1	0.93	−12.6
	65	1.01	0.77	−23.5	0.82	−19.2
	72	0.94	0.76	−19.6	0.81	−13.9
	90	0.96	0.88	−8.6	0.97	+1.0
			**Avg.**	−15.0	**Avg.**	−11.1
			**Total Avg.**	**−13.8**	**Total Avg.**	**−10.5**

**Table 4:** Theoretical and experimental values of the out-of-plane fracture angle for the U-notched spruce wood samples together with the percent discrepancies.

Notch Radius *ρ* (mm)	Loading Angle *β* (°)	Mean Experimental Fracture Angle (°)	MS Criterion		PS Criterion	
			Fracture Angle (°)	Fracture Angle (°)	Δ(%)	Δ(%)
1	40	24.6	22.4	22.2	−9.8	−9.0
	65	28.3	34.7	34.8	+23.1	+22.7
	72	35.4	39.0	39.0	+10.4	+10.3
	90	44.7	45.0	45.0	+0.7	+0.7
			**Avg.**	**Avg.**	+6.1	+6.1
2	40	16.3	19.0	18.9	+15.7	+16.1
	65	28.3	34.1	34.2	+20.6	+20.5
	72	36.0	39.0	39.0	+8.2	+8.2
	90	43.3	45.0	45.0	+3.8	+3.8
			**Avg.**	**Avg.**	+12.1	+12.2
4	40	19.3	19.9	19.0	−1.5	+3.0
	65	34.7	36.2	34.3	−0.9	+4.5
	72	39.7	39.2	39.0	−1.7	−1.2
	90	45.0	45.0	45.0	0.0	0.0
			**Avg.**	**Avg.**	−1.0	+1.5
			**Total Avg.**	**Total Avg.**	**+5.7**	**+6.6**

## Data Availability

The data presented in this study are available on request from the corresponding authors.

## Nomenclature

*E*	Elastic modulus
*υ*	Poisson’s ratio
*K_Ic_*	Mode I critical-stress-intensity factor
*K_IIIc_*	Mode III critical-stress-intensity factor
*ρ*	Notch tip radius
*β*	Loading angle
*σ_u_*	Ultimate tensile strength
*r_c,U_, d_c,U_*	Critical distances from the origin of the coordinate system
*σ_c_*	Critical stress of material
*K_I_^U,ρ^*	Mode I stress intensity factor for U-notch
*K_III_^U,ρ^*	Mode III stress intensity factor for U-notch
*A, A*,B, B*, C*, L, M, P, R, S, V, Z*	Constants
*ϕ_f_*	Out-of-plane fracture angle
*r*_0_	Distance between the notch tip and the origin of the coordinate system
*χ*_*b*1_, *χ*_*c*1_, *χ*_*d*1_	Notch parameters
*λ*_1_, *λ*_3_, *ω*_1_, *ω*_3_, *μ*_3_	Notch parameters
*M_U_^e^*	Mode mixity ratio
*K_eff_^U,ρ^*	Effective stress intensity factor for U-notch
